# CAR-Engineered NK Cells for the Treatment of Glioblastoma: Turning Innate Effectors Into Precision Tools for Cancer Immunotherapy

**DOI:** 10.3389/fimmu.2019.02683

**Published:** 2019-11-14

**Authors:** Michael C. Burger, Congcong Zhang, Patrick N. Harter, Annette Romanski, Florian Strassheimer, Christian Senft, Torsten Tonn, Joachim P. Steinbach, Winfried S. Wels

**Affiliations:** ^1^Institute for Neurooncology, Goethe University, Frankfurt am Main, Germany; ^2^Frankfurt Cancer Institute, Goethe University, Frankfurt am Main, Germany; ^3^German Cancer Consortium (DKTK), Partner Site Frankfurt/Mainz, Frankfurt am Main, Germany; ^4^German Cancer Research Center (DKFZ), Heidelberg, Germany; ^5^Georg-Speyer-Haus, Institute for Tumor Biology and Experimental Therapy, Frankfurt am Main, Germany; ^6^Neurological Institute (Edinger Institute), Goethe University, Frankfurt am Main, Germany; ^7^German Red Cross Blood Donation Service Baden-Württemberg–Hessen, Frankfurt am Main, Germany; ^8^Department of Neurosurgery, Goethe University, Frankfurt am Main, Germany; ^9^German Red Cross Blood Donation Service North-East, Dresden, Germany; ^10^Transfusion Medicine, Medical Faculty Carl Gustav Carus, Technical University Dresden, Dresden, Germany; ^11^German Cancer Consortium (DKTK), Partner Site Dresden, Dresden, Germany

**Keywords:** natural killer cells, NK-92, chimeric antigen receptor, adoptive cancer immunotherapy, glioblastoma

## Abstract

Glioblastoma (GB) is the most common and aggressive primary brain tumor in adults and currently incurable. Despite multimodal treatment regimens, median survival in unselected patient cohorts is <1 year, and recurrence remains almost inevitable. Escape from immune surveillance is thought to contribute to the development and progression of GB. While GB tumors are frequently infiltrated by natural killer (NK) cells, these are actively suppressed by the GB cells and the GB tumor microenvironment. Nevertheless, *ex vivo* activation with cytokines can restore cytolytic activity of NK cells against GB, indicating that NK cells have potential for adoptive immunotherapy of GB if potent cytotoxicity can be maintained *in vivo*. NK cells contribute to cancer immune surveillance not only by their direct natural cytotoxicity which is triggered rapidly upon stimulation through germline-encoded cell surface receptors, but also by modulating T-cell mediated antitumor immune responses through maintaining the quality of dendritic cells and enhancing the presentation of tumor antigens. Furthermore, similar to T cells, specific recognition and elimination of cancer cells by NK cells can be markedly enhanced through expression of chimeric antigen receptors (CARs), which provides an opportunity to generate NK-cell therapeutics of defined specificity for cancer immunotherapy. Here, we discuss effects of the GB tumor microenvironment on NK-cell functionality, summarize early treatment attempts with *ex vivo* activated NK cells, and describe relevant CAR target antigens validated with CAR-T cells. We then outline preclinical approaches that employ CAR-NK cells for GB immunotherapy, and give an overview on the ongoing clinical development of ErbB2 (HER2)-specific CAR-NK cells currently applied in a phase I clinical trial in glioblastoma patients.

## Introduction

Glioblastoma (GB) is the most frequent malignant primary brain tumor in adults, without any curative treatment options available at present. Patients diagnosed with GB have a dismal prognosis, and typically succumb to the disease within 3 months if untreated. Present standard of care for most patients includes surgical resection followed by radio- and chemotherapy. Despite this aggressive treatment, median survival of glioblastoma patients is only about 15 months, and recurrence remains almost inevitable (1, 2). Still <5% of patients diagnosed with GB survive more than 5 years (3). In contrast to other cancers such as adenocarcinomas of the lung or melanoma, primary brain tumors like GB and low grade gliomas (LGG) are known as rather immunologically “cold” tumors, typically with low numbers of tumor-infiltrating lymphocytes (TILs) (4), and the mere amount of TILs is not associated with patient survival (5). Nevertheless, the composition of the immunologic tumor microenvironment undergoes changes upon radiotherapy, chemotherapy, or even after anti-angiogenic therapy (6). Hence, intensifying efforts to overcome the general resistance of glioblastoma to immunotherapy appears highly warranted (7–9).

Recent clinical trials with chimeric antigen receptor (CAR)-engineered T cells demonstrated the feasibility and safety of this approach for the treatment of recurrent glioblastoma, with signs of clinical activity and transient responses observed in some of the patients (10–12). Efforts are underway to further refine these strategies, which includes evaluation of NK cells as alternative CAR-engineered effectors. The critical role of NK cells in cancer immune surveillance is increasingly being recognized (13, 14). NK cells do not only contribute to antitumor immunity by directly eliminating malignant cells, but also by regulating tumor-specific adaptive immune responses through crosstalk with dendritic cells (DCs) (15). NK cells thereby regulate DC maturation, and so determine the effectiveness of subsequent DC-mediated T-cell activation (16). Conversely, DCs enhance the direct antitumor activity of NK cells (17), which is also relevant for glioblastoma as shown in a recent phase II clinical trial with a dendritic cell vaccine (18). However, in cancer patients NK cells are often functionally compromised due to the immunosuppressive activity of the tumor. NK cells do not carry a T-cell receptor restricted to a particular peptide epitope presented by major histocompatibility complex (MHC) molecules, but recognize stress ligands on cancer cells via germ-line encoded activating receptors, which are counter-balanced by inhibitory receptors that are triggered by self-MHC class I. Hence, for adoptive cancer immunotherapy HLA-mismatched NK cells from healthy donors are preferred, which do not recognize tumor cells as “self,” thereby bypassing inhibitory signals. Since NK cells do not carry a high risk of inducing graft-vs.-host-disease (GvHD), this approach is generally considered to be safe (19–21). The better understanding of NK-cell biology, together with the development of strategies to enhance NK-cell activity by blocking inhibitory receptor pathways or redirect NK cells to tumors using bispecific antibodies or genetic modification with CARs, have paved the way for many therapeutic approaches that are now actively pursued in a clinical setting. These extend from the treatment of different types of leukemia to various solid tumors (22–24). Such refined approaches also hold enormous potential to improve immunotherapy of glioblastoma (25), assigning to NK cells a dual role as targeted killers and modulators of innate and adaptive immunity in the GB tumor microenvironment.

## NK Cells in the Tumor Microenvironment of Glioblastoma

Studies that evaluated infiltration of malignant gliomas by NK cells have reported different findings, ranging from the presence of insignificant NK cell numbers to large NK cell infiltrates in up to 89% of glioblastomas (26, 27). Another study described less infiltration of high-risk gliomas with NK cells and M1-like macrophages than lower grade gliomas (28). Therefore, differences in the subtype and stage of the disease, treatment history and permeability of the blood-brain barrier (BBB) appear to affect the likeliness of infiltration by NK cells. The presence of NK cells and their repertoire of activating killer cell immunoglobulin-like receptors (KIRs) can have an impact on disease progression. This was shown by Dominguez-Valentin et al. who analyzed 108 glioblastoma patients and 454 healthy individuals for HLA-A, -B, -C, NK-cell KIRs, and CMV-specific antibodies, and correlated the results with clinical parameters. The KIR allele KIR2DS4^*^00101 was thereby identified as an independent prognostic parameter of prolonged survival (29). Notably, all patients carrying KIR2DS4^*^00101 were CMV seropositive, and showed an increase in NK-cell subpopulations that expressed the cytotoxicity receptors CD16, NKG2D, and CD94/NKG2C. Healthy controls had a reduced risk to develop glioblastoma if they harbored two KIR2DS4^*^00101 alleles. Likewise, Gras Navarro et al. identified a subpopulation in donor-derived NK cells expressing KIR2DS2, which had a functional advantage in killing GB cells (30). Compared to KIR2DS2-negative NK cells, KIR2DS2-positive NK cells showed higher cytotoxicity, at least in part also mediated through expression of NKG2D ligands by the tumor cells. NKG2D-mediated control of glioblastoma by NK cells has also been demonstrated in experimental GB models (31).

Escape from immune surveillance is thought to contribute to the development and progression of GB (32, 33). GB tumors which are infiltrated by NK cells actively suppress NK-cell function via expression of factors such as transforming growth factor (TGF)-β, which is a main contributor to the immunosuppressive GB microenvironment (34, 35). TGF-β impairs NK cells by downregulating activating NK receptors such as NKG2D and NKp30 (36), or by repressing the mTOR pathway (37). This may be circumvented by making NK cells resistant to TGF-β, as recently achieved by expression of a dominant-negative TGF-β receptor in cord blood NK cells (38). Nevertheless, most GB cells also express high levels of MHC class I molecules, which inhibit autologous NK cells via inhibitory KIRs. Thus, blockade of such KIRs may favor a less tumor-promoting microenvironment and enhanced NK-cell mediated killing (25). GB cells can also affect NK-cell activity through expression of inhibitory molecules like regeneration and tolerance factor (RTF) and lectin-like transcript (LLT)-1 (33, 39), or indirectly through myeloid cells (40). Resident microglial cells in the brain are induced by glioma-derived TGF-β to acquire an immunosuppressive phenotype (41). Although the concept of M1 and M2 polarization of microglia in GB has been challenged as both subtypes are present in GB and prognostic relevance remains under debate (42), it is generally accepted that glioma-associated macrophages can reinforce an immunosuppressive tumor microenvironment (43, 44). Nevertheless, while actively suppressed in glioblastoma tumors, even patient-derived autologous NK cells can recover their cytolytic activity upon *ex vivo* culture with IL-2 or IL-15, in particular directed to GB stem-like cells (45, 46).

## Clinical Utility of NK Cells for the Treatment of Glioblastoma

Combinations of donor-derived NK cells with an antibody recognizing a glioblastoma-restricted surface antigen or a histone deacetylase inhibitor (HDACi) that induced upregulation of NKG2D ligand expression by GB cells have been shown in preclinical models to overcome immunosuppressive effects of brain tumors (47, 48). Also pre-treatment with the proteasome inhibitor bortezomib sensitized GB cells toward NKG2D- or TRAIL-mediated NK-cell lysis and enhanced survival in animal models (49). Available clinical data, however, are so far still restricted to earlier approaches based on *ex vivo* activated autologous immune cells. Ishikawa et al. performed a phase I clinical trial with autologous NK cells combined with systemic low-dose interferon (IFN)-β in nine patients with recurrent malignant glioma, including three patients with glioblastoma (50). NK cells were expanded from peripheral blood mononuclear cells (PBMCs) using irradiated feeder cells and IL-2. Repeated doses of NK cells were either only applied intravenously, or both, intravenously and directly into the tumor cavity through an Ommaya reservoir. The NK cell therapy proved to be safe and partially effective, with two patients experiencing a partial response (PR), two patients a mixed response (MR) and three patients stable disease (SD) during different courses of treatment (50).

The majority of early studies evaluating adoptive cell therapy, however, were performed with autologous lymphokine-activated killer (LAK) cells in combination with IL-2 injected into the resection cavity of recurrent or progressive malignant gliomas, with the cells usually applied through a reservoir. LAK cells are a mixture of T and NK cells derived by *ex vivo* culture of peripheral blood lymphocytes in IL-2-containing medium (51). Thereby the main lytic activity of LAK cells is mediated by CD3^−^CD56^+^ NK cells, while the contribution of CD3^+^CD56^−^ T cells is rather limited (52). The studies with LAK cells in glioma patients reported disease stabilization and partial or even complete responses in some of the patients, without encountering dose-limiting toxicities (53–64). Despite activation with IL-2, LAK cells can still be inhibited by immunosuppressive molecules secreted or presented by GB cells (65), which may explain why, despite the observed clinical activity in some cases, responses after therapy with autologous LAK cells were not durable. These reports are nevertheless encouraging and important for ongoing and future studies with enhanced NK-cell products such as CAR-NK cells, since they document feasibility and overall safety and tolerability of repeated intracavitary or intralesional injection of large numbers of lymphocytes together with IL-2, in some cases reaching up to 10^10^ cells per dose (55).

## The Concept of Chimeric Antigen Receptors

Chimeric antigen receptors were initially developed as a means for T cells to bypass MHC restriction of the T-cell receptor (TCR) and instead acquire TCR-independent, predetermined specificity for a defined cell surface antigen expressed by the target cell of interest (66–68). Since the first description of the basic CAR design encompassing a single chain fragment variable (scFv) antibody for target recognition linked to CD3ζ or FcεRIγ chains for signaling (first-generation CARs) (66), this approach has continuously been refined to enhance effector cell activity, and improve engraftment and persistence in the host upon adoptive transfer. Accordingly, receptors currently employed for CAR-T cell products approved for the treatment of malignancies of B-cell origin or undergoing clinical testing for various hematologic or solid tumor indications include in addition to CD3ζ one or more costimulatory protein domains, typically derived from CD28 and CD137 (4-1BB) (referred to as second- or third-generation CARs) (69, 70).

**Figure 1:**
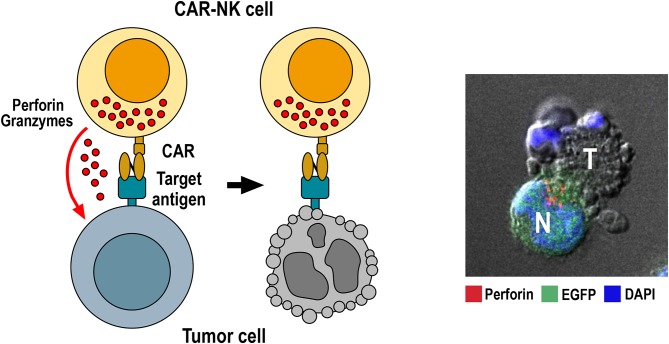
Killing of tumor cells by CAR-NK cells. Specific binding of the chimeric antigen receptor to its target antigen on the tumor-cell surface triggers CAR activation. This results in re-orientation of cytotoxic granules toward the immunological synapse formed between NK and target cell, followed by release of perforin and granzymes from cytotoxic granules into the synaptic cleft. Perforin and granzymes taken up by the target cell then trigger apoptotic cell death indicated by membrane blebbing and disintegration of the nucleus. The confocal microscopy image to the right shows conjugate formation between human GB cells co-expressing EGFR and EGFRvIII with NK-92/225.28.z CAR-NK cells that recognize both target antigens (71). Tumor (T) and EGFP-positive CAR-NK cells (N, green) were co-incubated for 1 h, fixed, permeabilized and stained for perforin (red) to identify cytotoxic granules. Cell nuclei were labeled with DAPI (blue).

Also NK cells can be genetically engineered to express chimeric antigen receptors, thereby acquiring in addition to their natural cytotoxicity built-in ADCC-like activity, similar to FcγRIIIa (CD16) activation by target-specific IgG molecules (Figure 1). This was first demonstrated for a CAR-like CD4-CD3ζ fusion receptor in human NK3.3 cells (72), and a scFv-CD3ζ-based first-generation CAR in NK-92 cells (73). Subsequently, peripheral blood NK cells from healthy donors, cord blood derived NK cells, and NK cells generated from induced pluripotent stem cells (iPSCs) have successfully been used for the generation of CAR-NK cells in preclinical studies (74–76). While conventional T-cell CARs with CD3ζ and CD28 and/or CD137 domains are functional in NK cells (74–80), several groups have reported improved activity if one or more signaling domains derived from CD244 (2B4), NKG2D, DAP10 or DAP12 were included in the receptors (75, 81, 82). It is presently unknown whether a particular CAR design would thereby be preferable for all CAR-NK cells irrespective of the nature of the cell binding domain, the target antigen, and the source and differentiation state of the effector cells. So far no CAR-NK cell product has received marketing authorization, but several early phase clinical trials in different cancer indications are ongoing, mainly based on genetically engineered NK-92 and cord blood NK cells (15, 23, 83).

## Glioblastoma Antigens Targeted in Clinical Trials With CAR-T Cells

Most studies on CAR-engineered effector cells in glioblastoma have so far focused on autologous CAR-T cells. These approaches have been reviewed extensively in recent years (84–88). Nevertheless, due to their relevance for NK-cell based therapies, some of their key findings are summarized below, concentrating in particular on tumor-associated antigens that have already been targeted in clinical trials.

## IL-13Rα2

Interleukin-13 receptor α 2 (IL-13Rα2) has first been described as a therapeutic target for GB two decades ago (89). It has been found overexpressed in >50% of high-grade gliomas, but is undetectable in normal brain tissue. IL-13Rα2 expression alone can induce invasiveness of GB cells without affecting cell growth, but together with the epidermal growth factor receptor variant EGFRvIII promotes GB cell proliferation (90). Initial CAR-T cell approaches targeting IL-13Rα2 employed IL-13 muteins for cell recognition in first-generation CARs (termed zetakines) to reduce binding to IL-13Rα1, which is not restricted to GB but broadly expressed in other tissues (91, 92). Later work demonstrated improved activity of such CAR-T cells *in vitro* and in orthotopic GB xenograft models if costimulatory protein domains were included in the CARs (93–96). To achieve exclusive binding to the target antigen, also scFv-based IL-13Rα2-specific CARs were developed, which eliminated remaining cross-reactivity to IL-13Rα1 (97–100). Safety and feasibility of local treatment of glioblastoma patients by repeated application of up to 10^8^ CD8^+^ T cells expressing a first-generation IL-13 zetakine into the resection cavity through a catheter and reservoir was demonstrated in a pilot trial at the City of Hope National Medical Center with three subjects, with signs of transient clinical activity observed in two patients. For one patient, tumor material before and after therapy was available, which indicated reduced IL-13Rα2 expression in the tumor after treatment (101). More recently, the same group reported on a patient with recurrent multifocal glioblastoma from an ongoing phase I clinical trial with autologous CAR-T cells carrying an improved second-generation CAR with an IL-13 mutein and CD137 and CD3ζ signaling domains (NCT02208362, clinicaltrials.gov) (10). The patient was treated with repeated infusions of up to 10^7^ CAR-T cells per dose into the resection cavity and the ventricular system. This resulted in the regression of intracranial and spinal tumors, with the clinical response continuing for 7.5 months after initiation of treatment. No dose-limiting toxicities were observed.

## EGFRvIII

Epidermal growth factor receptor (EGFR) is a well-established therapeutic target in glioblastoma (102). *EGFR* gene amplification and EGFR protein overexpression is present in 40 to 60% of GB tumors (103), while the receptor is not or only minimally expressed in normal brain tissue (104). Glioblastoma cells with *EGFR* gene amplification often co-express a constitutively active EGFR mutant form (EGFRvIII) (105, 106), which drives tumorigenicity and mediates radio- and chemoresistance (107, 108). *EGFR* gene amplification and expression of EGFRvIII are both correlated with poor prognosis and shorter survival of GB patients (109). *EGFRvIII* harbors an in-frame deletion of exons 2–7 of the wildtype *EGFR* gene. This generates a tumor-specific neo-epitope at the N-terminus of the receptor which can be targeted by specific immunotherapy. Consequently, several groups investigated EGFRvIII-targeted CAR-T cells that employed second- or third-generation CARs with scFv antibody domains specifically recognizing the EGFRvIII neo-epitope. These CAR-T cells demonstrated selective cytotoxicity against EGFRvIII-positive GB cells *in vitro* and potent activity against subcutaneous or orthotopic human GB xenografts in mice upon intravenous or intratumoral injection (110–115). In a phase I clinical trial at the University of Pennsylvania with 10 patients suffering from recurrent glioblastoma, intravenous application of a single dose of up to 5 × 10^8^ autologous EGFRvIII-specific CAR-T cells proved to be safe without evidence of off-tumor toxicity or cytokine release syndrome (12). One patient showed stable disease for over 18 months of follow-up. Transient expansion of CAR-T cells in the peripheral blood was found in all patients, and presence of CAR-T cells in GB tissue in five of seven patients where post-treatment tissue was available. While the level of *EGFR* gene amplification did not change, EGFRvIII expression had declined or was undetectable in the majority of such patients, which may have been due to the selective pressure exerted by the CAR-T cells (12). In a dose-escalation trial at the National Cancer Institute, 18 patients with recurrent EGFRvIII-positive glioblastoma were treated by intravenous infusion of up to ≥10^10^ autologous T cells carrying an EGFRvIII-specific third-generation CAR together with IL-2 after conditioning by lymphodepleting chemotherapy (116). One patient developed fatal and another patient serious respiratory symptoms shortly after cell infusion at the highest dose levels, which was attributed to congestion of pulmonary vasculature by activated T cells. No objective responses were observed, but one patient was still free from disease progression 6 months after therapy (116).

## ErbB2 (HER2)

Like EGFR, ErbB2 (HER2) is a member of the family of EGFR-related receptor tyrosine kinases. Activating mutations of ErbB2 appear less common than in the case of EGFR (117), but overexpression of the receptor is frequently found in breast carcinomas and many other epithelial cancers. Due to its ability to form signaling-competent heterodimers with all other members of the EGFR family, overexpression of ErbB2 strongly contributes to malignant transformation (118). While absent in the adult central nervous system (119), ErbB2 protein expression was found in up to 80% of GB tumors and was correlated with impaired survival (120–124). In a recent study with 56 primary GB tumors, immunohistochemical analysis revealed high ErbB2 expression in 21.5%, more moderate expression in another 19.6%, and no or low expression in 58.9% of the samples. Thereby, in the majority of cases ErbB2 expression in relapsed tumors was comparable to or higher than that of primary tumors from the same patients (125). In a preclinical study Ahmed et al. evaluated patient-derived CAR-T cells transduced with a retroviral vector encoding an ErbB2-specific CAR with CD28 and CD3ζ signaling domains. These CAR-T cells exhibited remarkable antitumor activity against ErbB2-positive autologous GB cells *in vitro* including CD133-positive stem-like cells derived from primary GB tumors, and in orthotopic GB xenograft models in mice (126). In a subsequent phase I dose-escalation trial at Baylor College of Medicine, the same group treated 17 patients with progressive ErbB2-positive glioblastoma by systemic infusion of one or more doses of up to 10^8^/m^2^ autologous ErbB2-specific CAR-T cells. The infusions were well-tolerated, without encountering dose-limiting toxicities. Of 16 evaluable patients, one had a partial response lasting 9 months, seven had stable disease, and eight progressed after CAR-T cell infusion. At the time of publication, three patients with stable disease were alive without evidence of progression 24 and 29 months after treatment (11).

## EphA2, CSPG4, CD133, and CD70

Other antigens that were proposed as targets for CAR-T cell approaches in glioblastoma are EphA2, CSPG4, CD133, and CD70. The receptor tyrosine kinase erythropoietin-producing hepatocellular carcinoma A2 (EphA2) is overexpressed in GB and contributes to its malignancy (127, 128). CAR-T cells carrying EphA2-specific second- or third-generation CARs displayed potent activity against glioma-initiating cells growing as neurospheres and orthotopic GB xenografts in murine models (129, 130). Chondroitin sulfate proteoglycan 4 (CSPG4) is highly expressed in different solid tumors including glioblastoma, where CSPG4 expression is associated with more aggressive disease. Of 46 tumor samples tested in a recent study, 67% were positive for CSPG4 (131). CAR-T cells with CSPG4-specific second- or third-generation CARs, among other cancer entities, also lysed CSPG4-positive glioblastoma cells, including GB stem-like cells (131–133). The CAR-T cells also effectively controlled the growth of orthotopic GB xenografts upon intracranial injection in mice, without selection of antigen-loss tumor cell variants. Interestingly, TNF-α released by tumor-associated microglia further induced CSPG4 expression in the GB cells (131). CD133 (prominin-1) is a stem cell marker, also expressed by cancer stem cells of many tumor entities including glioblastoma (134). Despite concerns of on-target/off-tumor toxicity against hematopoietic stem cells, the feasibility of systemic application of CD133-specific CAR-T cells with manageable toxicity was recently shown in a phase I clinical trial in patients with hepatocellular carcinoma and different epithelial cancers (135). In preclinical models, CAR-T cells expressing CD133-specific third-generation CARs with CD28, CD137, and CD3ζ signaling domains displayed specific cytotoxicity against patient-derived GB stem cells *in vitro* and in orthotopic GB mouse models (136, 137). CD70 is a member of the tumor necrosis factor (TNF) family and the ligand for CD27. While CD70 expression is typically restricted to highly activated T- and B-lymphocytes and a subset of mature DCs, certain hematologic malignancies and solid tumors, including gliomas, can constitutively overexpress CD70 (138, 139). Thereby, CD70 expression in GB cells directly facilitates immune evasion by selectively inducing CD8^+^ T-cell death (140). T cells expressing CD70-specific first- and second-generation CARs which utilized the CD70-binding domain of CD27 for target-cell recognition specifically lysed CD70-positive primary GB cells *in vitro*, and inhibited growth or induced complete tumor regression in xenograft and syngeneic GB models in mice (141). To reduce the risk of immune escape due to inhomogeneous target antigen expression, also CAR-T cell products were developed which simultaneously target two or three GB antigens. Strategies validated in GB xenograft models include the combination of individual CAR-T cells targeting ErbB2 or IL-13Rα2, or co-expression of ErbB2- and IL-13Rα2-specific CARs, or receptors targeting ErbB2, IL-13Rα2 and EphA2 in the same T cells (142, 143). Alternatively, an ErbB2-specific scFv antibody and an IL-13 mutein were directly combined in a single bispecific tandem CAR (TanCAR) (144).

## Advantages of CAR-NK Cells as Off-the-Shelf Therapeutics

Since autologous CAR-T cells are made from the cancer patients' own peripheral blood lymphocytes, yield, transduction efficiency, T-cell subtype distribution, and activation state can vary, affecting overall product composition and quality. In contrast, NK cells can safely be administered to an HLA-mismatched recipient. Hence, donor-derived peripheral blood and cord blood NK cells provide readily available resources not only for donor lymphocyte infusions of unmodified effector cells, but also for the generation of genetically engineered NK cells that may be provided as cost-effective off-the-shelf products (21, 76, 145). Furthermore, NK cells carry natural cytotoxicity receptors (NCRs) and the C-type lectin-like receptor NKG2D which are triggered rapidly by engagement of ligands selectively expressed by stressed and transformed cells (146). This natural cytotoxicity of NK cells can complement CAR-mediated cell killing, and may allow CAR-NK cells to also attack tumors with heterogeneous expression of the CAR target antigen (15). Nevertheless, because of the limited life span and expansion potential of primary NK cells, *ex vivo* culture for the generation of the required cell numbers can be demanding (147). Consequently, also human NK cell lines that continuously expand in the presence of IL-2 are being evaluated as an alternative source for the generation of well-defined clinical grade NK-cell products (148–150).

So far most of such efforts have focused on the NK-92 cell line, which was initially isolated from a non-Hodgkin lymphoma patient (151). NK-92 cells display features of activated primary NK cells and express many activating NK-cell receptors such as NKp30, NKp46, and NKG2D as well as high levels of granzymes A and B, but lack inhibitory NK-cell receptors except for KIR2DL4, Ig-like transcript 2 (ILT-2) and NKG2A/CD94 (151–155). General safety of repeated infusions of irradiated NK-92 cells at doses up to 10^10^ cells/m^2^ has been established in phase I clinical trials in patients with advanced cancers, with durable responses observed in some of the treated subjects (156–159). NK-92 cells were also instrumental to demonstrate that it is feasible to generate tumor-targeted CAR-NK cells for cancer immunotherapy. Since the initial description of this concept (73), the number of preclinical studies evaluating CAR-engineered NK-92 cells has steadily increased, demonstrating markedly enhanced antitumor activity of the cells if targeted to surface molecules expressed by different hematologic malignancies and solid tumors (15, 83), including tumor-associated antigens such as EGFR, EGFRvIII and ErbB2 that are relevant for the development of immunotherapies for glioblastoma (71, 125, 160) (outlined in the following section). In a recent phase I clinical trial with CD33-specific CAR NK-92 cells for the treatment of acute myeloid leukemia (AML), no dose-limiting toxicities were encountered upon repeated intravenous infusions of up to 5 × 10^9^ irradiated cells per dose (161), suggesting that the safety profile of these CAR NK-92 cells is similar to that of unmodified NK-92. Several other early phase clinical trials with CAR-engineered NK-92 cells are presently ongoing in Europe, China and the US (15, 83), including the CAR2BRAIN phase I clinical study which investigates a clonal ErbB2-specific CAR NK-92 product in glioblastoma patients and is described in more detail in a subsequent section (125, 162).

## Activity of EGFRvIII-Specific CAR-NK Cells in Preclinical Glioblastoma Models

The therapeutic utility of CAR-engineered NK cells for the treatment of glioblastoma has so far mainly been investigated in preclinical studies with effector cells targeting EGFRvIII, EGFR or ErbB2 (Table 1). Müller et al. generated a CAR based on a scFv fragment of EGFRvIII-specific antibody MR1-1 (166), which was fused to an intracellular DNAX-activating protein 12 (DAP12) domain for signaling (82). DAP12 contains an immunoreceptor tyrosine-based activation motif (ITAM), and like CD3ζ which harbors three ITAMs, it transmits signals from activating NK-cell receptors (167). The CAR was expressed in the established human NK cell line YTS, with the resulting effector cells showing enhanced lysis of GB cells transfected with an EGFRvIII construct or endogenously expressing the target antigen. Intravenous injection into mice carrying subcutaneous EGFRvIII-positive GB xenografts resulted in inhibition of tumor growth and extended survival, which was further enhanced by co-expressing the chemokine receptor CXCR4 in the CAR-NK cells for improved tumor homing (82). Enhanced cytotoxicity against EGFRvIII-expressing GB cells was also found in *in vitro* cell culture assays with established human KHYG-1 NK cells expressing an EGFRvIII-specific CAR (163). In a study with CAR-engineered NK-92 cells, a second-generation chimeric antigen receptor was used that employed EGFRvIII-specific antibody MR1-1 as a cell binding domain and CD28 and CD3ζ domains for signaling (71). Upon contact formation, the respective CAR-NK cells re-oriented their cytotoxic granules toward EGFRvIII-positive but not EGFRvIII-negative GB cells, resulting in rapid and selective tumor cell killing. High and specific cytotoxicity of these CAR-NK cells was also demonstrated with targets growing in a tissue-like environment using patient-derived primary colon cancer organoids transduced with an EGFRvIII-encoding vector as a model (168). Repeated stereotactic injection of the EGFRvIII-specific NK-92 cells into orthotopic EGFRvIII-positive GB xenografts in immunodeficient NSG mice delayed tumor growth and markedly improved symptom-free survival (71). However, treatment of mixed tumors which similar to the clinical situation consisted of EGFR and EGFRvIII double-positive, and EGFR-expressing but EGFRvIII-negative GB cells, resulted in a less pronounced survival benefit and selective outgrowth of EGFRvIII-negative tumors still expressing the wildtype receptor. This is reminiscent of EGFRvIII-negative tumor recurrence observed in some GB patients after treatment with EGFRvIII-specific CAR-T cells (12).

**Table 1:** Preclinical studies with CAR-NK cells in brain cancer models.

**Target**	**Antibody**	**Hinge**	**TM**	**Signaling**	**Effector cells**	**Gene transfer**	**Cancer type**	***In vivo* model**	**Treatment**	**Reference**
EGFRvIII	MR1-1	Myc-tag	DAP12	DAP12	YTS	Lentivirus	GB	s.c. xenografts in NMRI nude mice	i.v. injection	(82)
EGFRvIII	MR1-1	CD8α	CD28	CD28- CD3ζ	NK-92	Lentivirus	GB	orthotopic xenografts in NSG mice	i.t. injection	(71)
EGFRvIII	3C10	CD8α	CD28	CD28-CD137-CD3ζ	KHYG-1	Lentivirus	GB	−	−	(163)
EGFR	R1	CD8α	CD28	CD28- CD3ζ	NK-92	Lentivirus	GB	orthotopic xenografts in NSG mice	i.t. injection	(71)
EGFRvIII and EGFR	528	n.s.	CD28	CD28- CD3ζ	NK-92 NKL	Lentivirus	GB	orthotopic xenografts in NSG mice	i.t. injection	(160)
EGFRvIII and EGFR	Cetuximab (225)	CD8α	CD28	CD28- CD3ζ	NK-92	Lentivirus	GB	orthotopic xenografts in NSG mice	i.t. injection	(71)
ErbB2 (HER2)	FRP5	CD8α	CD3ζ	CD3ζ	NK-92	Retrovirus	Breast ca. brain metastasis	orthotopic xenografts in athymic nude rats	i.v. injection with FUS	(164, 165)
ErbB2 (HER2)	FRP5	CD8α	CD28	CD28- CD3ζ	NK-92	Lentivirus	GB	orthotopic xenografts in NSG mice	i.t. injection	(125)
ErbB2 (HER2)	FRP5	CD8α	CD28	CD28- CD3ζ	NK-92	Lentivirus	GB	syngeneic orthotopic tumors in C57BL/6 mice	i.t. injection	(15, 125)

*TM, transmembrane domain; s.c., subcutaneous; i.v., intravenous; i.t., intratumoral; n.s., not specified; GB, glioblastoma; Breast ca., breast carcinoma; FUS, MRI-guided focused ultrasound*.

## CAR-NK Cells Simultaneously Targeting EGFR and EGFRvIII

To circumvent the problem of heterogeneous EGFRvIII expression in GB tumors, two groups generated CAR-NK cells which simultaneously target wildtype EGFR and the receptor variant. Han et al. employed as a cell binding domain of a second-generation CAR with CD28 and CD3ζ signaling domains a scFv fragment of EGFR-specific antibody 528, which recognizes an epitope conserved in EGFRvIII (160). Established human NK-92 and NKL cells expressing this CAR displayed enhanced cytotoxicity and IFN-γ secretion when cocultured with GB cell lines or patient-derived GB stem cells expressing EGFR or EGFRvIII. Treatment of NSG mice carrying orthotopic GB xenografts expressing wildtype EGFR or EGFRvIII by repeated intracranial injection of CAR-engineered NK-92 cells inhibited tumor growth more strongly than treatment with NK-92 control cells and improved overall survival of the animals. In an independent study, Genßler et al. used a scFv fragment derived from the clinically applied antibody cetuximab as a cell binding domain for a dual-specific second-generation CAR with CD28 and CD3ζ signaling domains (71). Like antibody 528, chimeric antibody cetuximab and its murine parent 225 recognize an epitope common to EGFR and EGFRvIII (169, 170). The MR1-1-based CAR outlined above which is specific for the EGFRvIII neo-epitope and a similar CAR based on antibody R1 that recognizes an N-terminal epitope only present in wildtype EGFR were included in this study for comparison. *In vitro* analysis of NK-92 cells carrying these CARs revealed high and specific cytotoxicity of EGFR-targeted effectors against established and primary human GB cells, which was dependent on EGFR expression and CAR signaling. Cytotoxicity of EGFRvIII-targeted NK-92 was restricted to EGFRvIII-positive GB cells, while dual-specific NK cells were active against tumor cells positive for EGFR and/or EGFRvIII (Figure 1). Importantly, in NSG mice carrying orthotopic GB xenografts xenografts either expressing EGFR, EGFRvIII or both receptors, repeated local treatment with the dual-specific NK cells was superior to treatment with either one or a mixture of the corresponding monospecific CAR-NK cells. This led to a marked extension of survival without inducing the rapid immune escape of antigen-loss variants that was observed upon therapy with monospecific CAR-NK cells (71). In later work, Jiang et al. followed a similar strategy to co-target EGFR and EGFRvIII with CAR-T cells recognizing a shared epitope of the receptors (171). Co-targeting of EGFR and EGFRvIII has also been achieved by expressing a secreted EGFR-specific T-cell engager in EGFRvIII CAR-T cells (172). Nevertheless, due to the more limited life span and expansion potential of NK cells when compared to T cells, utilizing CAR-NK cells may be a safer approach for targeting a surface antigen such as wildtype EGFR which is highly expressed also by vital normal tissues.

## Preclinical Evaluation of CAR-NK Cells Specific for ErbB2 (HER2)

In addition to EGFRvIII and EGFR, the receptor tyrosine kinase ErbB2 has been targeted with CAR-engineered NK-92 cells. The respective NK-92/5.28.z cells represent a molecularly and functionally well-defined single cell clone isolated upon transduction of NK-92 cells under GMP-compliant conditions with a lentiviral vector encoding a second-generation CAR very similar to the one used by Ahmed et al. for CAR-T cells (11), based on ErbB2-specific antibody FRP5 and a composite CD28-CD3ζ signaling domain (78, 173). In preclinical studies, these cells displayed high and selective cytotoxicity against ErbB2-positive target cells of different solid tumor origins including established GB cell lines, and primary GB stem cell cultures (78, 125). Importantly, specific cytotoxicity of NK-92/5.28.z against GB cells was retained under hypoxic conditions and in the presence of high concentrations of immunosuppressive TGF-β, indicating that the cells remain functional in an environment similar to that of a GB tumor. Systemic application in NSG mice resulted in selective enrichment of NK-92/5.28.z cells in orthotopic breast carcinoma xenografts and a reduction of pulmonary tumor nodules in an experimental renal cell carcinoma metastasis model (78). In orthotopic GB xenograft models, repeated stereotactic injection of the cells into the tumor area effectively inhibited tumor progression, and led to a marked extension of survival (125). Furthermore, in an immunocompetent GB mouse model, NK-92/5.28.z cells displayed strong immunomodulatory activity and enhanced endogenous antitumor immunity upon intratumoral injection, resulting in tumor rejection in the majority of mice carrying syngeneic intracranial GL261/ErbB2 glioblastomas. In contrast, unmodified parental NK-92 cells were unable to inhibit tumor progression (15, 125). Without further treatment, those mice that were cured from their initial tumors also rejected a rechallenge with the GB cells injected into the other brain hemisphere more than 120 days after initial therapy, indicating induction of a long-lasting protective immune response induced in the animals by treatment with NK-92/5.28.z. IgG antibodies in the sera of these mice were broadly directed against the GB cells and not limited to the CAR target antigen. Protective immunity induced by initial treatment with NK-92/5.28.z cells was also dependent on T-cell memory, since depletion of CD4^+^ and CD8^+^ T cells before rechallenge prevented tumor rejection in some of the animals (15). Because of these promising preclinical data, the ErbB2-specific NK-92/5.28.z CAR-NK cells were chosen for further development toward clinical application in glioblastoma patients (described in the following sections).

## CAR-NK Cells Targeting CD133 and GD_2_

Also NK-92 cells engineered to express a CAR specific for the tumor-associated antigen and GB target CD133 have been investigated, but preclinical data are so far limited to *in vitro* assays with CD133-positive established and primary ovarian carcinoma cells, which were eliminated by the CAR-NK cells when combined with cisplatin (174). In addition, the disialoganglioside GD_2_ is considered a potential target for glioblastoma therapy (175, 176). While GD_2_-specific CAR-NK cells have so far not been tested in GB models, both, primary donor-derived NK cells and NK-92 cells expressing GD_2_-specific first- or second-generation CARs demonstrated selective antitumor activity in preclinical *in vitro* and *in vivo* models of neuroblastoma, melanoma, breast carcinoma and Ewing sarcoma (81, 177–179).

## Manufacturing of CAR-NK-92 Cells for Clinical Application

As outlined above, the use of an NK cell line such as NK-92 offers certain advantages over primary NK cells which can overcome hurdles like variable transduction efficiency of viral vectors and limited expansion potential, that can complicate clinical translation of donor-derived NK cells (15). On the downside, due to their origin from a non-Hodgkin lymphoma, there may be a risk of secondary lymphoma formation in treated patients, which is currently addressed by including γ-irradiation of NK-92 before transfusion into a recipient (156–159, 161). Preclinical titration experiments demonstrated that an irradiation dose of 10 Gy is sufficient to inhibit proliferation of unmodified NK-92 cells as well as CAR-engineered NK-92/5.28.z, without affecting their *in vitro* cytotoxicity for at least 48 h or reducing *in vivo* antitumor activity in murine models (78, 125, 148, 180). This has of course implications for the manufacturing strategy. Autologous CAR-T cell products can be frozen after transduction and will expand *in vivo* upon injection of relatively small amounts of the cells. In contrast, NK cells in general and especially NK cell lines need to be expanded to a therapeutic dose before infusion. Irradiation will prevent any *in vivo* expansion, which mandates repeated therapeutic dosages for continued control of cancer growth. Hence, it would be ideal to produce cryopreserved cell doses in advance that can be thawed before transfusion. However, NK cells represent large granular lymphocytes which are more sensitive to cryopreservation than T cells, usually displaying impaired viability and long lag phases after thawing (181). While efforts are made to improve cryopreservation of NK cells, and some NK-92 cell products under commercial development are provided in cryopreserved form (154, 182, 183), we decided to use fresh NK-92/5.28.z cells in exponential growth phase for application in glioblastoma patients.

Adapting the strategy developed for unmodified NK-92 cells (148, 157), first a qualified Master Cell Bank (MCB) of CAR-engineered NK-92/5.28.z cells was generated, providing a reliable source for subsequent production of patient doses for clinical trials (180). To bridge the lag phase of cell proliferation after thawing of vials from the MCB and to have a therapeutic dose of NK-92/5.28.z cells readily available for a patient within <1 week, a process was established which relies on a maintenance culture of the CAR NK-92 cells. From this maintenance culture therapeutic dosages can be expanded in batch culture within 5 to 6 days upon seeding the cells at an initial density of 5 × 10^4^ cells/mL in 2 L of medium in gas permeable cell culture bags. After approximately 3.5 doublings (doubling time of NK-92/5.28.z: 32–36 h), the culture yields a total cell number of 1 × 10^9^, which is sufficient to supply several cell dosages comprising 1 × 10^8^ cells, which is the highest dose level currently planned for GB patients (180). Quality control performed on the maintenance culture involves monthly in-process controls for sterility, mycoplasma, endotoxin, absence of replication-competent lentivirus (p24 antigen ELISA), viability, identity, and phenotype (CD56^+^, CD16^−^, CAR^+^), as well as potency of the NK-92/5.28z cells (lysis of ErbB2-positive target cells) (180). Release criteria of the irradiated finished cell product comprise the same tests, but with the results of sterility testing available only after injection due to the required time period. Special care was taken to choose an excipient used as injection solution for intracranial injection of CAR-NK cells that would not elicit adverse reactions due to calcium or electrolyte imbalances. Accordingly, the NK-92/5.28.z cells are resuspended in human serum derived from a single blood donor. Using this approach, a shelf life of up to 22 h at 4 ± 2°C was established, which allows preparation and release of the investigational medicinal product 1 day before scheduled injection into a patient. The irradiated cell product is transferred to the clinical site in a bag comprising cells at a density of 5 × 10^7^ cells/mL. The neurosurgeon then transfers the required amount of this cell suspension into a syringe at the site of operation to dilute the CAR-NK cells depending on the dose level and to perform the intracranial injections. Our experience shows that this approach is feasible and provides a ready-to-use dosage of CAR-NK cells to the application site without the need for local processing (thawing, washing, cell counting), which reduces the risk for secondary contaminations. Nevertheless, the limited shelf life demands an orchestrated and well-organized process involving all partners.

## The CAR2BRAIN Phase I Clinical Trial

**Figure 2:**
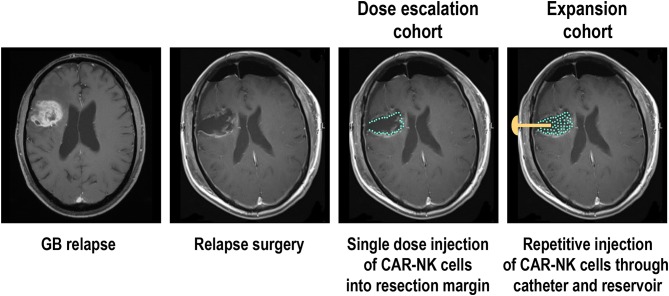
Phase I clinical trial with ErbB2-specific CAR NK-92 cells in glioblastoma patients. Patients with recurrent ErbB2-positive glioblastoma in the dose escalation cohort of the CAR2BRAIN phase I clinical trial (NCT03383978, clinicaltrials.gov) receive a single treatment with irradiated ErbB2-specific NK-92/5.28.z cells injected into the resection margin during relapse surgery. In the subsequent expansion cohort, patients are scheduled to receive in addition up to 12 weekly treatments applied into the resection cavity through an implanted catheter and reservoir. MRI images are for illustration only and do not show an actual patient treated in the study.

The phase I clinical trial CAR2BRAIN (NCT03383978, clinicaltrials.gov) is an investigator-initiated, prospective, open-label study carried out at the Center for Neurology and Neurosurgery at the University Hospital Frankfurt, Frankfurt am Main, Germany in patients with recurrent or refractory ErbB2-positive glioblastoma with a scheduled relapse surgery. Initially conducted as a single-center trial, it is now planned to include several other participating study centers in Germany. Main objective of the trial is to evaluate safety and tolerability of ErbB2-specific NK-92/5.28.z CAR-NK cells (78, 180) and to determine the maximum tolerated dose (MTD) or maximum feasible dose (MFD). In the dose-escalation cohort of the CAR2BRAIN study, NK-92/5.28.z cells are injected into the wall of the resection cavity during relapse surgery (Figure 2). The CAR-NK cells are subjected to γ-irradiation at a dose of 10 Gy prior to injection as part of the manufacturing process (see above). The dose levels explored are 1 × 10^7^, 3 × 10^7^, and 1 × 10^8^ NK-92/5.28.z cells used as a single dose application in a total injection volume of 2 mL, distributed by the neurosurgeon with multiple injections. Patients are enrolled in cohorts of three to six patients per dose level in a 3 + 3 dose escalation scheme. Up to now we concluded the first two dose levels, with evaluation of the third and last dose level ongoing. So far, no dose-limiting toxicities were encountered.

Direct injection of the CAR-NK cells into the wall of the resection cavity was chosen to allow for high effector cell density in the tumor and the infiltration zone. Unlike ErbB2-specific autologous CAR-T cells that were applied systemically in GB patients in a recent phase I dose-escalation trial (11), irradiated NK-92/5.28.z CAR-NK cells cannot engraft permanently or expand *in vivo*, considerably limiting the number of viable cells that may reach the brain tissue from the circulation. Systemically applied NK-92/5.28.z cells homed to orthotopically implanted breast carcinoma xenografts in mice and were effective against experimental metastasis irrespective of γ-irradiation (78). However, intravenously injected CAR NK-92 cells had no therapeutic effect against intracranial tumors in preclinical studies, suggesting that at least in rodent models they cannot cross the blood-brain barrier in sufficient numbers without applying measures such as focused ultrasound (164, 165). This may be different in GB patients where the BBB can be severely disturbed in large parts of the tumor (184), and apparently allows systemically applied CAR-T cells access to the cancer cells (11, 12). Nevertheless, encouraging data on the successful treatment of a glioblastoma patient from a phase I clinical trial by local injection of IL-13Rα2-specific CAR-T cells suggest that intracranial delivery of effector cells may indeed be the preferred route of administration (10).

While in the case of B-cell malignancies a single dose of CAR-T cells is often sufficient to achieve durable responses (69, 70), repeated application can be more beneficial for the therapy of solid tumors, and will likely be mandatory to induce a lasting response if treatment consists of irradiated effector cells such as NK-92 (15, 150). Accordingly, after concluding dose escalation in the CAR2BRAIN trial, it is planned to include additional patients in an expansion cohort, scheduled for multiple injections of the CAR-NK cells at the recommended safe dose established during dose escalation (Figure 2). In these patients, intraoperative injection of NK-92/5.28.z cells is followed by implantation of a Rickham reservoir, with the catheter of the reservoir placed in the resection cavity. Beginning 1 week after surgery and intra-operative treatment with CAR-NK cells, patients will receive up to 12 additional injections of NK-92/5.28.z into the resection cavity, applied through the reservoir and catheter. With these treatments spaced 1 week apart, resident GB cells can be exposed to NK-92/5.28.z for a prolonged time period while no potentially problematic dose accumulation of the irradiated effector cells is expected. In addition to the analysis of peripheral blood scheduled at different time points—if possible—also cerebrospinal fluid (CSF) will be collected through the catheter before each treatment for analysis of soluble factors and cells contained therein, expected to provide insights on possible effects of the CAR-NK cells on endogenous immune cells over the course of therapy. Patients will also be tested for potential immune responses to the allogeneic NK-92 cells and the CAR construct. In different phase I clinical trials with parental NK-92 cells, an anti-HLA antibody response to NK-92 was reported in one out of two, one out of seven, and six out of twelve patients evaluated (156, 157, 159). It is presently unknown to what extent such responses can affect the activity of NK-92 cells during repeated dosing.

## Conclusions and Future Perspectives

NK-cell based adoptive immunotherapy of cancer is a rapidly expanding field. Recent advances such as the identification and effective blocking of distinct NK-cell immune checkpoints, and tumor-specific redirection of the exquisite lytic capacity of these innate lymphocytes using bispecific killer-cell engagers or chimeric antigen receptors can be expected to benefit patients suffering from many hematologic malignancies and solid tumors. Thereby glioblastoma, due to its location in the brain, its aggressiveness and highly immunosuppressive tumor microenvironment represents a particular challenge. Following similar approaches with CAR-T cells, CAR-NK cells have now entered clinical development for the treatment of malignant glioma. In addition to ongoing work with CAR-engineered NK-92 cells, other allogeneic off-the-shelf therapeutics based on donor-derived peripheral blood or cord blood NK cells, or NK cells differentiated from iPSCs may be tested for their effectiveness against brain tumors in the near future. However, overcoming inhibitory mechanisms in GB, and addressing immune escape due to inhomogeneous expression of CAR target antigens remain significant obstacles. While currently limited to a few cases, analysis of glioblastoma tissues from patients before and after treatment with CAR-T cells suggests that high effector cell activity can result in rapid selection of antigen-loss variants (12, 101). A similar problem may be encountered with CAR-NK cells. Nevertheless, NK cells naturally exhibit broad cytotoxicity triggered by interaction of their activating receptors with stress ligands expressed by tumor cells, which is retained by CAR-NK cells and may help to eliminate glioblastoma cells with low or absent expression of the CAR target antigen. Stem-like GB cells in particular appear sensitive to this natural cytotoxicity of allogeneic NK cells (185). Also preclinical data with NK-92 cells point in this direction, where ErbB2-specific CAR NK-92 cells lysed ErbB2-positive stem-like GB cells growing as neurospheres quite rapidly, but after prolonged exposure, the target cells were also eliminated by parental NK-92 cells lacking the CAR (125). The shorter life span and limited *in vivo* expansion of NK cells, while providing increased safety when compared to CAR-T cells, will likely restrict their long-term effectiveness and require repeated treatment. Ectopic expression of pro-inflammatory cytokines such as IL-15 together with the CAR may not only increase persistence of the CAR-NK cells themselves (76, 77), but also support T-cell infiltration and activation (186). Induction of endogenous immunological memory by CAR-NK cell treatment has been identified as the basis for cures in immunocompetent animal models of glioblastoma (15, 125). In GB patients, avoidance of prophylactic dexamethasone administration (187), and rational combination of adoptive NK-cell transfer with radiotherapy, immune checkpoint inhibitors, inhibitors of angiogenesis or modulators of the myeloid cell compartment may all be effective strategies to fully realize the cytotoxic potential of CAR-NK cells against glioblastoma (6, 49, 188, 189), and further enhance their ability to modulate innate and adaptive immune cells in the tumor microenvironment.

## Author Contributions

All authors made substantial contributions to the conception, design and writing of this review article, critically evaluated the cited literature, revised the manuscript, and approved the final version of this study. All authors agreed to be accountable for the content of this work.

### Conflict of Interest

CZ, TT, and WW are named as inventors on patents and patent applications in the field of cancer immunotherapy owned by their respective institutions. The remaining authors declare that the research was conducted in the absence of any commercial or financial relationships that could be construed as a potential conflict of interest.
